# On Ulam's Type Stability of the Cauchy Additive Equation

**DOI:** 10.1155/2014/540164

**Published:** 2014-03-16

**Authors:** Janusz Brzdęk

**Affiliations:** Department of Mathematics, Pedagogical University, Podchorążych 2, 30-084 Kraków, Poland

## Abstract

We prove a general result on Ulam's type stability of the functional equation *f*(*x* + *y*) = *f*(*x*) + *f*(*y*), in the class of functions mapping a commutative group into a commutative group. As a consequence, we deduce from it some hyperstability outcomes. Moreover, we also show how to use that result to improve some earlier stability estimations given by Isaac and Rassias.

## 1. Introduction

The issue of stability of functional equations has been a very popular subject of investigations for the last nearly fifty years (see, e.g., [1–8]). Its main motivation was given by Ulam (cf. [9–11]) in 1940 in his talk at the University of Wisconsin. For instance, we can introduce the following definition, which somehow describes the main ideas of such stability notion for equations in two variables (ℝ_+_ stands for the set of nonnegative reals).

**Definition 1**

Let *A* be a nonempty set, (*X*, *d*) be a metric space, *𝒞* ⊂ ℝ_+_^*A*^2^^ be nonempty, *𝒯* be an operator mapping *𝒞* into ℝ_+_^*A*^, and *ℱ*_1_, *ℱ*_2_ be operators mapping nonempty *𝒟* ⊂ *X* ^*A*^ into *X* ^*A*^2^^. We say that the equation

(1) $$\begin{array}{c} {ℱ}_{1}\varphi \left(x,y\right)={ℱ}_{2}\varphi \left(x,y\right) \end{array}$$

is *𝒯*-stable provided for every *ε* ∈ *𝒞* and *φ*_0_ ∈ *𝒟* with

(2) $$\begin{array}{c} d\left({ℱ}_{1}\varphi_{0}\left(x,y\right),{ℱ}_{2}\varphi_{0}\left(x,y\right)\right)\leq \varepsilon \left(x,y\right)\;x,y\in A, \end{array}$$

there exists a solution *φ* ∈ *𝒟* of (1) such that

(3) $$\begin{array}{c} d\left(\varphi \left(x\right),\varphi_{0}\left(x\right)\right)\leq 𝒯\varepsilon \left(x\right)\;x\in A. \end{array}$$

(As usual, *C* ^*D*^ denotes the family of all functions mapping a set *D* ≠ *∅* into a set *C* ≠ *∅*.) Roughly speaking, *𝒯*-stability of (1) means that every approximate (in the sense of (2)) solution to (1) is always close (in the sense of (3)) to an exact solution to (1). The next theorem is an example of the most classical results.

**Theorem 2**

Let *E*_1_ and *E*_2_ be two normed spaces and let *c* ≥ 0 and *p* ≠ 1 be fixed real numbers. Let *f* : *E*_1_ → *E*_2_ be an operator such that

(4) ||f(x+y)−f(x)−f(y)||  ≤c(||x||p+||y||p) x,y∈E1∖{0}.

If *p* ≥ 0 and *E*_2_ is complete, then there is a unique operator *T* : *E*_1_ → *E*_2_ that is additive (i.e., *T*(*x* + *y*) = *T*(*x*) + *T*(*y*) for *x*, *y* ∈ *E*_1_) and such that

(5) $$\begin{array}{c} ||f\left(x\right)-T\left(x\right)||\leq \frac{c{||x||}^{p}}{\left|2^{p-1}-1\right|}\;x\in E_{1}\setminus \left\{0\right\}. \end{array}$$

If *p* < 0, then *f* is additive.

It has been motivated by Rassias (see [12–14]) and is composed of the outcomes in [15–17]. Note that Theorem 2 with *p* = 0 yields the result of Hyers [9] and it is known (see [17]; cf. also [18, 19]) that for *p* = 1 an analogous result is not valid. Moreover, it has been shown in [20] that estimation (5) is optimum for *p* ≥ 0 in the general case.

The second statement of Theorem 2, for *p* < 0, can be described as the *φ*-hyperstability of the additive Cauchy equation for *φ*(*x*, *y*) ≡ *c*(||*x*||^*p*^ + ||*y*||^*p*^) (for further information on hyperstability see, e.g., [1, 16, 21, 22]; some other recent results can be found in [23–25]). It seems to be of interest that such result does not remain valid if we restrict the domain of *f* to a subsemigroup of the group (*E*_1_, +). The subsequent remark shows this.

**Remark 3**

Let *p* < 0, *a* ≥ 0, *I* = (*a*, *∞*), and *f*, *T* : *I* → ℝ be given by *T*(*x*) = 0 and *f*(*x*) = *x* ^*p*^ for *x* ∈ *I*. Then clearly

(6) $$\begin{array}{c} \left|f\left(x\right)-T\left(x\right)\right|=x^{p}\;x\in I. \end{array}$$

Moreover,

(7) $$\begin{array}{c} \left|f\left(x+y\right)-f\left(x\right)-f\left(y\right)\right|\leq x^{p}+y^{p}\;x,y\in I. \end{array}$$

In fact, suppose, for instance, that *x* ≤ *y*. Then (*x* + *y*)^*p*^ ≤ (2*x*)^*p*^ = 2^*p*^ *x* ^*p*^ ≤ *x* ^*p*^ ≤ *x* ^*p*^ + *y* ^*p*^, whence |*f*(*x* + *y*) − *f*(*x*) − *f*(*y*)| = *x* ^*p*^ + *y* ^*p*^ − (*x*+*y*)^*p*^ ≤ *x* ^*p*^ + *y* ^*p*^.

In this paper we prove a quite general result that allows us to generalize and extend Theorem 2 in various directions.

## 2. An Auxiliary Result

In the proof of the main theorem in this paper, we use the following fixed point result that can be easily derived from [26, Theorem 2] (cf. [27, Theorem 1] and [28]). For a survey on applications of the fixed point methods for similar issues, see [29].

**Theorem 4**

Assume that *Z* is a nonempty set, (*Y*, *d*) is a complete metric space, *f*_1_, *f*_2_ : *Z* → *Z*, *𝒯* : *Y* ^*Z*^ → *Y* ^*Z*^ is an operator satisfying the inequality

(8) d(𝒯ξ(x),𝒯μ(x))≤d(ξ(f1(x)),μ(f1(x)))+d(ξ(f2(x)),μ(f2(x))),ξ,μ∈YZ, x∈Z,

and Λ : ℝ_+_^*Z*^ → ℝ_+_^*Z*^ is an operator defined by

(9) $$\begin{array}{c} \Lambda \delta \left(x\right):=\delta \left(f_{1}\left(x\right)\right)+\delta \left(f_{2}\left(x\right)\right)\;\delta \in {\mathbb{R}}_{+}^{Z},\;\;x\in Z. \end{array}$$

Suppose that there exist functions *ε* : *Z* → ℝ_+_ and *φ* : *Z* → *Y* such that

(10) d(𝒯φ(x),φ(x))≤ε(x),ε∗(x):=∑n=0∞(Λnε)(x)<∞ x∈Z,

where Λ^*n*^ denotes the *n*th iterate of Λ (i.e., Λ^0^ *δ* = *δ* for *δ* ∈ ℝ_+_^*Z*^ and Λ^*n*^ : = Λ ○ Λ^*n*−1^ for *n* ∈ *ℕ*). Then there exists a unique fixed point *ψ* of *𝒯* with

(11) $$\begin{array}{c} d\left(\varphi \left(x\right),\psi \left(x\right)\right)\leq \varepsilon^{*}\left(x\right)\;x\in Z. \end{array}$$

Moreover,

(12) $$\begin{array}{c} \psi \left(x\right):=\underset{n\to \infty }{\lim}\left({𝒯}^{n}\varphi \right)\left(x\right)\;x\in Z. \end{array}$$

## 3. The Main Theorem

Given a group (*X*, +), we denote by *Aut* *X* the family of all automorphisms of *X*. Moreover, for each *u* ∈ *X* ^*X*^ we write *ux* : = *u*(*x*) for *x* ∈ *X* and we define *u*′ ∈ *X* ^*X*^ by *u*′*x* : = *x* − *ux*.

The next theorem is the main result of this paper.

**Theorem 5**

Let (*X*, +) and (*E*, +) be commutative groups, *d* be a complete metric in *E* that is invariant (i.e., *d*(*x* + *z*, *y* + *z*) = *d*(*x*, *y*) for *x*, *y*, *z* ∈ *X*), *H* : (*X*∖{0})^2^ → ℝ_+_, and

(13) l(X):={u∈Aut  X:u′∈Aut  X,  λ(u′)+λ(u)<1}≠∅,

where

(14) λ(u)≔inf⁡{t∈ℝ+:H(ux,uy)   ≤tH(x,y)  ∀x,y∈X∖{0}}

for *u* ∈ *Aut* *X*. Assume that *f* : *X* → *E* satisfies the inequality

(15) $$\begin{array}{c} d\left(f\left(x+y\right),f\left(x\right)+f\left(y\right)\right)\leq H\left(x,y\right)\;x,y\in X\setminus \left\{0\right\}. \end{array}$$

Then, for each nonempty *𝒰* ⊂ *l*(*X*) such that

(16) $$\begin{array}{c} u\;○\;v=v\;○\;u\;u,v\in 𝒰, \end{array}$$

there exists a unique additive *T* : *X* → *E* fulfilling the inequality

(17) $$\begin{array}{c} d\left(f\left(x\right),T\left(x\right)\right)\leq H_{𝒰}\left(x\right)\;x\in X\setminus \left\{0\right\}, \end{array}$$

where

(18) $$\begin{array}{c} H_{𝒰}\left(x\right):=\inf\left\{\frac{H\left(u^{\prime }x,ux\right)}{1-\lambda \left(u\right)-\lambda \left(u^{\prime }\right)}:u\in 𝒰\right\}\;x\in X\setminus \left\{0\right\}. \end{array}$$

**Proof**

Let *𝒰* ⊂ *l*(*X*) be nonempty and let (16) be valid. Write *X*_0_ : = *X*∖{0}. Note that (15), with *x* replaced by *u*′*x* and *y* = *ux*, gives

(19) d(f(x),f(u′x)+f(ux))  ≤H(u′x,ux) x∈X0,  u∈𝒰.

Given *u* ∈ *𝒰*, we define operators *𝒯*_*u*_ : *E* ^*X*_0_^ → *E* ^*X*_0_^ and Λ_*u*_ : ℝ_+_^*X*_0_^ → ℝ_+_^*X*_0_^ by

(20) $$\begin{array}{c} {𝒯}_{u}\xi \left(x\right):=\xi \left(u^{\prime }x\right)+\xi \left(ux\right)\;x\in X_{0},\;\;\xi \in E^{X_{0}},\;\;u\in 𝒰, \end{array}$$

(21) $$\begin{array}{c} \;\Lambda_{u}\delta \left(x\right):=\delta \left(u^{\prime }x\right)+\delta \left(ux\right)\;x\in X_{0},\;\;\delta \in {\mathbb{R}}_{+}^{X_{0}},\;\;u\in 𝒰. \end{array}$$

It is easily seen that, for each *u* ∈ *𝒰*, Λ : = Λ_*u*_ has form (9) with *Z* : = *X*_0_, *f*_1_(*x*) = *u*′*x*, and *f*_2_(*x*) = *ux*. Moreover, (19) can be written in the following way

(22) d(f(x),𝒯uf(x))≤H(u′x,ux)=:εu(x) x∈X0,  u∈𝒰.

(Here and in the sequel, the restriction of *f* to the set *X*_0_ is also denoted by *f*; we believe that this will not cause any confusion.) And

(23) d(𝒯uξ(x),𝒯uμ(x))  =  d(ξ(u′x)+ξ(ux),μ(u′x)+μ(ux))  ≤  d(ξ(u′x),μ(u′x))+d(ξ(ux),μ(ux))

for every *ξ*, *μ* ∈ *E* ^*X*_0_^, *x* ∈ *X*_0_, and *u* ∈ *𝒰*. Consequently, for each *u* ∈ *𝒰*, also (8) is valid with *Z* : = *X*_0_, *Y* : = *E*, and *𝒯* : = *𝒯*_*u*_.

Note that, in view of the definition of *λ*(*u*),

(24) $$\begin{array}{c} H\left(ux,uy\right)\leq \lambda \left(u\right)H\left(x,y\right)\;u\in 𝒰,\;\;x,y\in X_{0}. \end{array}$$

So, it is easy to show by induction on *n* that

(25) $$\begin{array}{c} \Lambda_{u}^{n}\varepsilon_{u}\left(x\right)\leq {\left(\lambda \left(u^{\prime }\right)+\lambda \left(u\right)\right)}^{n}H\left(u^{\prime }x,ux\right), \end{array}$$

for *x* ∈ *X*_0_, *n* ∈ *ℕ*_0_ (nonnegative integers), and *u* ∈ *𝒰*. Hence,

(26) εu∗(x):∑n=0∞(Λunεu)(x)≤  H(u′x,ux)∑n=0∞(λ(u′)+λ(u))n=  H(u′x,ux)1−λ(u)−λ(u′) x∈X0,  u∈𝒰.

Now, we can use Theorem 4 with *Z* = *X*_0_, *Y* = *E*, *ε* : = *ε*_*u*_, and *φ* = *f*. According to it, the limit

(27) $$\begin{array}{c} T_{u}^{\prime }\left(x\right):=\underset{n\to \infty }{\lim}\left({𝒯}_{u}^{n}f\right)\left(x\right) \end{array}$$

exists for each *x* ∈ *X*_0_ and *u* ∈ *𝒰*,

(28) $$\begin{array}{c} d\left(f\left(x\right),T_{u}\left(x\right)\right)\leq \frac{H\left(u^{\prime }x,ux\right)}{1-\lambda \left(u\right)-\lambda \left(u^{\prime }\right)}\;x\in X_{0},\;\;u\in 𝒰, \end{array}$$

and the function *T*_*u*_ : *X* → *E* defined by

(29) $$\begin{array}{c} T_{u}\left(0\right)=0,\;\;T_{u}\left(x\right):=T_{u}^{\prime }\left(x\right)\;x\in X_{0} \end{array}$$

is a solution of the equation

(30) $$\begin{array}{c} \;T\left(x\right)=T\left(u^{\prime }x\right)+T\left(ux\right), \end{array}$$

because *T*_*u*_′ is a fixed point of *𝒯*_*u*_.

Now we show that

(31) d(𝒯unf(x+y),𝒯unf(x)+𝒯unf(y))  ≤(λ(u′)+λ(u))nH(x,y)

for every *x*, *y* ∈ *X*_0_, *x* + *y* ≠ 0, *n* ∈ *ℕ*_0_, and *u* ∈ *𝒰*.

Since the case *n* = 0 is just (15), take *k* ∈ *ℕ*_0_ and assume that (31) holds for *n* = *k* and every *x*, *y* ∈ *X*_0_, *x* + *y* ≠ 0, and *u* ∈ *𝒰*. Then, by (24),

(32) d(𝒯uk+1f(x+y),𝒯uk+1f(x)+𝒯uk+1f(y))  =d(𝒯ukf(u′(x+y))+𝒯ukf(u(x+y)),     𝒯ukf(u′x)+𝒯ukf(ux)+𝒯ukf(u′y)     +𝒯ukf(uy))  ≤  d(𝒯ukf(u′x+u′y),𝒯ukf(u′x)+𝒯ukf(u′y))   +d(𝒯ukf(ux+uy),𝒯ukf(ux)+𝒯ukf(uy))  ≤(λ(u′)+λ(u))kH(u′x,u′y)   +(λ(u′)+λ(u))kH(ux,uy)  ≤(λ(u′)+λ(u))k+1H(x,y)x,y∈X0, x+y≠0, u∈𝒰.

Thus, by induction, we have shown that (31) holds for every *x*, *y* ∈ *X*_0_, *x* + *y* ≠ 0, *n* ∈ *ℕ*_0_, and *u* ∈ *𝒰*. Letting *n* → *∞* in (31), we obtain the equality

(33)   Tu(x+y)=Tu(x)+Tu(y) x,y∈X0, x+y≠0, u∈𝒰.

From this we can deduce that *T*_*u*_ is additive for each *u* ∈ *𝒰*. The reasoning is very simple, but for the convenience of readers we present it here.

In view of (33), it is only enough to consider the situation *y* = −*x*. So take *u* ∈ *𝒰* and *x* ∈ *X*_0_ (the case *x* = 0 is trivial). Then, by (33),

(34) Tu(x)=Tu(x+x−x)=Tu(2x)+Tu(−x)=2Tu(x)+Tu(−x),

which yields *T*_*u*_(*x*) + *T*_*u*_(−*x*) = 0 and consequently *T*_*u*_(*x* − *x*) = *T*_*u*_(0) = 0 = *T*_*u*_(*x*) + *T*_*u*_(−*x*).

Next, we prove that each additive *T* : *X* → *Y* satisfying the inequality

(35) $$\begin{array}{c} \;d\left(f\left(x\right),T\left(x\right)\right)\leq LH\left(vx,v^{\prime }x\right)\;x\in X_{0}, \end{array}$$

with some *L* > 0 and *v* ∈ *𝒰*, is equal to *T*_*w*_ for each *w* ∈ *𝒰*. To this end fix *v*, *w* ∈ *𝒰*, *L* > 0, and an additive *T* : *X* → *Y* satisfying (35). Note that, by (28) and (35), there is *L*_0_ > 0 such that

(36) d(T(x),Tw(x))≤d(T(x),f(x))+d(f(x),Tw(x))≤L0(H(v′x,vx)+H(w′x,wx))×∑n=0∞(λ(w′)+λ(w))n

for *x* ∈ *X*_0_. Observe yet that *T* and *T*_*w*_ are solutions to (30) for all *u* ∈ *𝒰*, because they are additive.

We show that, for each *j* ∈ *ℕ*_0_,

(37) d(T(x),Tw(x))  ≤L0(H(v′x,vx)+H(w′x,wx))   ×∑n=j∞(λ(w′)+λ(w))n x∈X0.

The case *j* = 0 is exactly (36). So fix *l* ∈ *ℕ*_0_ and assume that (37) holds for *j* = *l*. Then, in view of (24),

(38) d(T(x),Tw(x))  =d(T(wx)+T(w′x),Tw(wx)+Tw(w′x))  ≤  d(T(wx),Tw(wx))+d(T(w′x),Tw(w′x))  ≤  L0(H(v′wx,vwx)+H(w′wx,wwx))   ×∑n=j∞(λ(w′)+λ(w))n   +L0(H(v′w′x,vw′x)+H(w′w′x,ww′x))   ×∑n=j∞(λ(w′)+λ(w))n  ≤  L0(H(v′x,vx)+H(w′x,wx))(λ(w)+λ(w′))   ×∑n=j∞(λ(w′)+λ(w))n  =  L0(H(v′x,vx)+H(w′x,wx))   ×∑n=l+1∞(λ(w′)+λ(w))n x∈X0.

Thus we have shown (37). Now, letting *j* → *∞* in (37), we get

(39) $$\begin{array}{c} \;T\left(x\right)=T_{w}\left(x\right)\;x\in X_{0}. \end{array}$$

Since *T* and *T*_*w*_ are additive, we have *T* = *T*_*w*_.

In this way, we also have proved that *T*_*u*_ = *T*_*w*_ for each *u* ∈ *𝒰* (on account of (28)), which yields

(40) $$\begin{array}{c} \;d\left(f\left(x\right),T_{w}\left(x\right)\right)\leq \frac{H\left(u^{\prime }x,\;ux\right)}{1-\lambda \left(u^{\prime }\right)-\lambda \left(u\right)}\;x\in X_{0},\;\;u\in 𝒰. \end{array}$$

This implies (17) with *T* : = *T*_*w*_; clearly, equality (39) means the uniqueness of *T*, as well.

Thus we have completed the proof of Theorem 5.

## 4. Some Consequences

Theorem 5 yields the subsequent corollary.

**Corollary 6**

Let *X*, *H*, *E*, and *d* be as in Theorem 5. Suppose that there exists a nonempty *𝒰* ⊂ *l*(*X*) such that (16) holds and

(41) $$\begin{array}{c} \underset{u\in 𝒰}{\inf}\;H\left(u^{\prime }x,ux\right)=0\;x\in X\setminus \left\{0\right\},\\ \;\underset{u\in 𝒰}{\sup}\;\lambda \left(u^{\prime }\right)+\lambda \left(u\right)<1. \end{array}$$

Then every *f* : *X* → *E* satisfying (15) is additive.

**Proof**

Suppose that *f* : *X* → *E* satisfies (15). Then, by Theorem 5, there exists an additive *T* : *X* → *E* such that (17) holds. Since, in view of (41), *H*_*𝒰*_(*x*) = 0 for *x* ∈ *X*∖{0}, this means that *f*(*x*) = *T*(*x*) for *x* ∈ *X*∖{0}, whence

(42) $$\begin{array}{c} \;f\left(x+y\right)=f\left(x\right)+f\left(y\right)\;x,y\in X\setminus \left\{0\right\},\;\;x+y\neq 0, \end{array}$$

which implies that *f* is additive (see the proof of (34)).

The next corollary corresponds to the results on the inhomogeneous Cauchy equation (44) in [30–35].

**Corollary 7**

Let *X*, *H*, *E*, and *d* be as in Theorem 5 and *F* : *X* ^2^ → *E*. Suppose that

(43) $$\begin{array}{c} d\left(F\left(x,y\right),0\right)\leq H\left(x,y\right)\;x,y\in X, \end{array}$$

*F*(*x*_0_, *y*_0_) ≠ 0 for some *x*_0_, *y*_0_ ∈ *X*∖{0}, and there exists a nonempty *𝒰* ⊂ *l*(*X*) such that (16) and (41) hold. Then the inhomogeneous Cauchy equation

(44) $$\begin{array}{c} f\left(x+y\right)=f\left(x\right)+f\left(y\right)+F\left(x,y\right) \end{array}$$

has no solutions in the class of functions *f* : *X* → *E*.

**Proof**

Suppose that *f* : *X* → *E* is a solution to (44). Then

(45) d(f(x+y),f(x)+f(y))  =  d(f(x)+f(y)+F(x,y),f(x)+f(y))  =  d(F(x,y),0)≤H(x,y) x,y∈X∖{0}.

Consequently, by Corollary 6, *f* is additive, whence *F*(*x*_0_, *y*_0_) = *f*(*x*_0_ + *y*_0_) − *f*(*x*_0_) − *f*(*y*_0_) = 0, which is a contradiction.

**Remark 8**

We have excluded *x* = 0 and *y* = 0 from the domain of *H*, in Theorem 5, because of the reason which can be easily deduced from the subsequent natural example.

In the rest of this paper, we assume that *E*_1_ and *E* are normed spaces, *X* is a subgroup of the group (*E*_1_, +), with *d*(*x*, *y*) = ||*x* − *y*||, and *H* : (*X*∖{0})^2^ → ℝ_+_. For each *n* ∈ *ℤ* define *μ*_*n*_ : *X* → *X* by *μ*_*n*_ *x* : = *nx* for *x* ∈ *X*. Let *H* be given by

(46) $$\begin{array}{c} H\left(x,y\right)=c{||x||}^{p}+d{||y||}^{q}\;x,y\in X\setminus \left\{0\right\} \end{array}$$

with some real *p* < 0, *q* < 0, *c* ≥ 0, and *d* ≥ 0. Then

(47) H(μnx,μky)=H(nx,ky)=c||nx||p+d  ||ky||q=c|n|p||x||p+d  |k|q||y||q≤(|n|p+|k|q)(c||x||p+d||y||q)=(|n|p+|k|q)H(x,y)

for every *x*, *y* ∈ *X*∖{0}, *k*, *n* ∈ *ℤ*, and *kn* ≠ 0. Hence,

(48) lim⁡n→∞H(μnx,μn′y)  ≤lim⁡n→∞(np+(n−1)q)H(x,y)  =0 x,y∈X

and there is *M* > 1 such that

(49) $$\begin{array}{c} \lambda \left(\mu_{n}\right)+\lambda \left(\mu_{n}^{\prime }\right)=n^{p}+{\left(n-1\right)}^{q}<\frac{1}{2}\;n\in \mathbb{N},\;\;n>M. \end{array}$$

So, it is easily seen that conditions (41) are fulfilled with

(50) $$\begin{array}{c} 𝒰:=\left\{\mu_{n}\in Aut\;X:n\in \mathbb{N},n>M\right\} \end{array}$$

and therefore (by Corollary 6) every *f* : *X* → *E* satisfying (15), with *H* given by (46) is additive.

Clearly, the above reasoning also works (after an easy modification) when the function *H* : (*X*∖{0})^2^ → ℝ_+_ has the following a bit more involved form

(51) $$\begin{array}{c} \;H\left(x,y\right)=c{||\eta \left(x\right)||}^{p}+d{||\chi \left(y\right)||}^{q}\;x,y\in X\setminus \left\{0\right\}, \end{array}$$

with some real *p* < 0, *q* < 0, *c* ≥ 0, and *d* ≥ 0 and additive injections *η*, *χ* : *X* → *E*_1_ (or *η*, *χ* : *X* → *E*). So, we have the following corollary corresponding to the hyperstability results in [16, 21, 24] (see also [1, 22, 23, 25]).

**Corollary 9**

Let *H* be given by (51) with some real *p* < 0, *q* < 0, *c* ≥ 0, and *d* ≥ 0 and some additive injections *η*, *χ* : *X* → *E*_1_ (*η*, *χ* : *X* → *E*, resp.). Then every *f* : *X* → *E* satisfying (15) is additive.

We also get an analogous conclusion when *H* is given by

(52) $$\begin{array}{c} \;H\left(x,y\right)=c{||\eta \left(x\right)||}^{p}{||\chi \left(y\right)||}^{q}\;x,y\in X\setminus \left\{0\right\}, \end{array}$$

with some real *c* > 0 and *p*, *q* ∈ ℝ such that *p* + *q* < 0 and some additive injections *η*, *χ* : *X* → *E*_1_ (or *η*, *χ* : *X* → *E*), because

(53) H(nx,ky)=c||η(nx)||p||χ(ky)||q=c|n|p||η(x)||p|k|q||χ(y)||q

for every *x*, *y* ∈ *X*∖{0}, *k*, *n* ∈ *ℤ*, and *kn* ≠ 0. So we have the following hyperstability result, as well (it generalizes to some extend the main outcome in [36]).

**Corollary 10**

Let *H* be given by (52) with some *p*, *q* ∈ ℝ, *p* + *q* < 0, *c* ≥ 0, and some additive injections *η*, *χ* : *X* → *E*_1_ (*η*, *χ* : *X* → *E*, resp.). Then every *f* : *X* → *E* satisfying (15) is additive.

It is easily seen that another example of the function *H* satisfying (41) is given by

(54) $$\begin{array}{c} H\left(x,y\right)={\left(c{||\eta \left(x\right)||}^{p}+d\;{||\chi \left(y\right)||}^{q}\right)}^{r},\;x,y\in X\setminus \left\{0\right\}, \end{array}$$

with some real *p* > 0, *q* > 0, *r* < 0, *c* ≥ 0, *d* ≥ 0, *c* + *d* > 0 and some additive injections *η*, *χ* : *X* → *E*_1_ (*η*, *χ* : *X* → *E*, resp.), because

(55) H(nx,ky)=(c|n|p||η(x)||p+d  |k|q||χ(y)||q)r≤(min⁡⁡{|n|p,|k|q})r(c||η(x)||p+d||χ(y)||q)r=(min⁡⁡{|n|p,|k|q})rH(x,y)

for every *x*, *y* ∈ *X*∖{0}, *k*, *n* ∈ *ℤ*, and *kn* ≠ 0. So, we have yet the following.

**Corollary 11**

Let *H* be given by (54) with some real *p* > 0, *q* > 0, *r* < 0, *c* ≥ 0, *d* ≥ 0, *c* + *d* > 0 and some additive injections *η*, *χ* : *X* → *E*_1_ (*η*, *χ* : *X* → *E*, resp.). Then every *f* : *X* → *E* satisfying (15) is additive.

We finish the paper with an example of corollary that generalizes some results in [37] and improves the estimations obtained there.

**Corollary 12**

Let *X* be divisible by 2 and let *H* be given by (51) with some real numbers 1 < *p* < *q*, *c* ≥ 0, and *d* ≥ 0 and some additive injections *η*, *χ* : *X* → *E*_1_ (*η*, *χ* : *X* → *E*, resp.). Then, for every *f* : *X* → *E* satisfying (15), there exists an additive mapping *T* : *X* → *E* such that

(56) $$\begin{array}{c} ||f\left(x\right)-T\left(x\right)||\leq \frac{c{||\eta \left(x\right)||}^{p}+2^{p-q}d\;{||\chi \left(y\right)||}^{q}}{2^{p}-2},\;x\in X.\; \end{array}$$

**Proof**

Let *f* : *X* → *E* satisfy (15) and *u*_0_ : *X* → *X* be given by

(57) $$\begin{array}{c} u_{0}\left(x\right)=\frac{1}{2}x\;x\in X. \end{array}$$

Then *u*_0_ = *u*_0_′ and *λ*(*u*_0_) ≤ 2^−*p*^. Consequently, by Theorem 5 with *𝒰* = {*u*_0_}, there is a unique additive *T* : *X* → *E* such that

(58) ||f(x)−T(x)||≤c||η(u0x)||p+d||χ(u0′y)||q1−2λ(u0)≤2−pc||η(x)||p+2−q  d  ||χ(y)||q1−21−p=c||η(x)||p+2p−qd||χ(y)||q2p−2 x∈X.
